# Sexuality and childbearing as it is experienced by women living with HIV in Sweden: a lifeworld phenomenological study

**DOI:** 10.1080/17482631.2018.1487760

**Published:** 2018-07-04

**Authors:** Ewa Carlsson-Lalloo, Marie Berg, Åsa Mellgren, Marie Rusner

**Affiliations:** a Institute of Health and Care Sciences, Sahlgrenska Academy, University of Gothenburg, Gothenburg, Sweden; b Clinic of Infectious Diseases, Södra Älvsborg Hospital, Borås, Sweden; c Department of Research, Södra Älvsborg Hospital, Borås, Sweden

**Keywords:** HIV, women, sexuality, reproduction, health, phenomenology

## Abstract

The effectiveness of antiretroviral treatment has reduced sexual HIV transmission and mother-to-child-transmission. To optimally support women living with HIV, health care providers need deepened knowledge about HIV, sexuality and childbearing. The aim of this study was to describe the phenomenon sexuality and childbearing as experienced by women living with HIV in Sweden. Data were collected by phenomenon-oriented interviews with 18 HIV-positive women. A reflective lifeworld analysis based on phenomenological philosophy was conducted, describing the meaning structure of the phenomenon. The essence of the phenomenon is that perceptions about HIV and its contagiousness profoundly influence sexual habits and considerations in relation to pregnancy and childbearing. These perceptions are formed in combination with knowledge and interpretations about HIV by the women themselves and by their environments. The essence is further described by its constituents: Risk of transmission imposes demands on responsibility; The contagiousness of HIV limits sexuality and childbearing; Knowledge about HIV transmission provides confident choices and decisions; and To re-create sexuality and childbearing. Although HIV has a low risk of transmission if being well treated, our study shows that HIV-positive women feel more or less contagious, which influences sexuality and decision-making in relation to become pregnant and give birth.

## Introduction

Sexuality and childbearing are integral parts of health and being a woman and, by ensuring that women have access to sexual and reproductive health services with good quality, these elements can also contribute to fulfilling and strengthening women’s human rights (World Health Organization [WHO], 2013). Women living with HIV have important, specific sexual and reproductive health-related needs (Patterson et al., 2017). Increased access to antiretroviral treatment (ART) and starting early with ART after diagnosis has, together with the effectiveness of ART, reduced mortality and morbidity in HIV-positive people (Nakagawa et al., 2012; Van Sighem, Gras, Reiss, Brinkman, & De Wolf, 2010). Therefore, HIV infection is today considered a chronic disease in well treated individuals. The effectiveness of ART has also reduced the risk of transmission to a sexual partner (Cohen et al., 2011; Wawer et al., 2005), a risk which is even questioned to be possible when an individual is well treated with ART (Rodger et al., 2016). ART during pregnancy, suppressing plasma HIV RNA, and mothers refraining from breastfeeding the baby have also been found to significantly reduce the risk of mother-to-child-transmission (MTCT) to a level below 0.5% in well treated individuals (Mandelbrot et al., 2015; Swedish Reference Group for Antiviral Therapy [RAV], 2017).

Globally, 36.7 million people are estimated to be HIV-positive and more than half of them are women above the age of 15 years (WHO, 2016). It is estimated that 77% of HIV-positive pregnant women receive ART, with varied coverage between different countries (from 2% to > 95%) (Joint United Nations Programme on HIV/AIDS [UNAIDS], 2017). In Sweden, in 2016, 95% of the 6983 people diagnosed with HIV (≈0.07% of Sweden’s total population [Statistics, 2018]) were well treated with ART (plasma HIV RNA < 50 copies/ml) (InfCareHIV, 2016) and had a variety of ages and origins (40% women, 60% men) (Marrone, Mellgren, Eriksson, Svedhem & De Socio, 2016). Around 60–80 children are born yearly to women living with HIV in Sweden (≈0.05–0.07% of all babies born in Sweden in 2013 [Statistics, 2018]) with an MTCT rate < 0.5% (Smittskyddsinstitutet [SMI], 2013). HIV infection is legally regulated in Sweden by the Communicable Diseases Act, which stipulates that people living with HIV must always use a condom in sexual encounters and that they must inform a sexual partner about their HIV diagnosis, unless they are well treated (Sveriges Riksdag, 2004). As a mother, a woman must not breastfeed her baby (SMI, 2013). In 2013–2014, due to the new knowledge about low risk of sexual transmission, the interpretation of the Swedish law changed, which made it possible for the treating physician to remove the HIV-positive person’s duty to inform about HIV in sexual encounters in instances where they have not been diagnosed with other sexually transmitted diseases, they demonstrate good adherence to ART, and they have undetectable plasma viral load (HIV RNA < 50 copies/ml) (Albert et al., 2014; SMI, 2013).

Beyond medical care, health care providers have to consider social and psychological needs to help patients with HIV to attain better health, including their sexual and reproductive health (Kumar, Gruskin, Khosla, & Narasimhan, 2015). Although there has been increased access to ART, studies examining sexuality and reproduction for women living with HIV still show that HIV impacts sexuality and reproduction negatively (Carter et al., 2017a; Greene, Ion, Kwaramba, Smith, & Loutfy, 2015; Rahangdale et al., 2014). Women with HIV therefore face unique challenges, such as in partner relationships, sexual satisfaction and childbearing (Bharat & Mahendra, 2007; Carter et al., 2013; Florence et al., 2004). Many women living with HIV have a desire to give birth to a child (Carter et al., 2017b; Wessman et al., 2015), a desire which can further increase feelings of stigmatization, shame and fear (Sandelowski & Barroso, 2003; Sanders, 2008). In a meta-synthesis of 18 qualitative studies, it was demonstrated that HIV was a burden in relation to sexuality and reproduction for HIV-positive women, and that the burden comprises fear of transmitting HIV to a partner or infant (Carlsson-Lalloo, Rusner, Mellgren, & Berg, 2016). In 2017, a Swedish inductive interview study examining seven HIV-positive women’s sexuality showed that fear of transmitting HIV to one’s partner is highly present (Norwald, Holmström, & Plantin, 2017). In order to support the health and well-being of women living with HIV, health care professionals need access to a solid knowledge base about issues related to HIV, sexuality and childbearing (Carter et al., 2017a; Shapiro & Ray, 2007). There is still insufficient knowledge regarding what sexuality and childbearing means to women living with HIV in a Swedish context. Therefore, the aim of this study was to describe the phenomenon sexuality and childbearing as it is experienced by women living with HIV in Sweden.

## Methods

The phenomenon sexuality and childbearing, as it is experienced by women living with HIV in Sweden, was explored by using the reflective lifeworld approach based on phenomenological philosophy as described by Dahlberg, Dahlberg, and Nyström (2008).

The lifeworld can be explained as a world with meanings, where people experience and share the world in relation to each other. There is no thinking that is separate from the body, but the body, subject and the world are intertwined. The so-called intersubjective world is accessed through the lived body, which is embedded and manifests itself through the lived experiences (Dahlberg et al., 2008; Merleau-Ponty, 2012).

The reflective lifeworld approach contains methodological principles as openness, flexibility and bridling (Lindberg, Österberg, & Höreberg, 2016). According to Dahlberg et al. (2008), bridling is a methodological principle where the researchers need to embody a phenomenological attitude, which means adopting an openness and flexibility towards the explored phenomenon. It is a reflective attitude which aims to slow down the process of understanding as a whole, making what is not directly visible become visible. It includes restraining one’s pre-understanding and avoiding the act of defining what is undefinable (Dahlberg & Dahlberg, 2003) is. This is fundamental for research validity and transferability in studies with such a design (Todres, Galvin, & Dahlberg, 2007).

### Participants

The inclusion criteria were: women living with HIV-diagnosis, aged >18 years, who spoke English or Swedish and lived in the western region of Sweden. Exclusion criteria were: women with newly diagnosed HIV-infection (within 6 months), or/and with ongoing crisis reaction or serious mental illness. The participants were strategically chosen to present women living with HIV in Sweden and also to reflect a wide variety of the participants’ experiences of the explored phenomenon. Women of a variety of ages, years living with HIV, time living in Sweden, having a partner or not, cultural background, or experience of being a mother were considered when choosing the participants. Five clinics in the region participated and women meeting the inclusion criteria were invited to participate in the study and received oral and written information, either by a nurse, medical counsellor or physician. Women interested in participating in the study were contacted by the first author and were given a more comprehensive description of the purpose of the study. A total of 23 women expressed interest in participating in the study. Five of them were not interviewed due to the following reasons: two women did not show up for the meeting, two changed their mind regarding participation and one woman did not want the interview to be recorded. Thus, 18 women from 3 HIV clinics were included. The age range was 30–60 years and the participants had been diagnosed with HIV between the years 1992 and 2015. The women represented nine different countries; nine participants were born in an African country, seven were born in Sweden and two were born in Asia. The women not born in Sweden had lived in Sweden for different periods of time. Their family status varied, including whether they had a partner (13 women stated that they had a partner, some partners with HIV and some without HIV) and whether they had children or had ever been pregnant.

### Data collection

Individual phenomenon-oriented interviews were conducted between September 2015 and April 2016 by the first author in a setting chosen by the participant. Three of the interviews were conducted in English so as not to exclude the women who had not lived in Sweden for a longer period of time. The interviews lasted between 42 and 101 minutes. The interviews began with a broad open question: What is it like to live with HIV? The focus during the interview was to gain access to personal experiences about the phenomenon, *sexuality and childbearing as it is experienced by women living with HIV*, and questions such as “How do you experience your sexuality?” and “What is it like to be pregnant and living with HIV?” were asked. In order to encourage reflection and to develop richer illustrations of the experiences of the phenomenon, follow-up questions such as “Can you please give an example of that experience?” and “How did that make you feel?” were asked. The interviews were digitally recorded and transcribed verbatim: the Swedish interviews in Swedish and the English interviews in English.

The analysis included both Swedish and English material. The quatations were translated by the first author and that translation was verified by the other bilingual authors. The translated text was then reviewed for language by a native English speaker for grammar and spelling.

### Phenomenological lifeworld analysis

A phenomenological lifeworld analysis, as described by Dahlberg et al. (2008), was conducted to describe the meaning structure of the phenomenon. The description of the phenomenon, the meaning structure, is a unity of its invariant part, the essence, and its variances of the essence, the so-called constituents.

The analysis follows a process which begins with the whole, then analyses its parts and, finally, reconstructs the whole to understand the meaning structure of the phenomenon. The first step was therefore to conduct a thorough reading of the transcribed interviews in order to obtain a sense of the whole data material and to become acquainted with the material. Next, the attention was directed towards parts in the text to further uncover particular nuances of meaning. Meaning units—a word, a sentence, or a longer piece of text, relevant to experiences of the phenomenon, sexuality and childbearing—were identified. The computer program NVivo 11 (QSR International Pty Ltd., 2016) was used to organize, but not to analyse, the data material. Meanings within these units were described with a few words and were then compared, to identify differences and similarities. The meaning units that were considered to be similar, or related in other ways, were grouped into clusters as a preliminary analysing stage, on the way to elucidating the essence of the phenomenon. At this level the researchers used bridling during the continuing process of discovery, reflecting and working through the meanings, and trying to understand each meaning as a figure against the background of the others. The bridling allowed the researchers to slow down the process of understanding the phenomenon, and, in this way, allow its meanings to show themselves. When the meanings relevant to the phenomenon had been identified and no inconsistencies could be found, the essence emerged through a movement between the parts and the whole. The essence was expressed and described and, as a final stage, the variances of the essence that were present in the data were then further described in the more contextual nuances of the phenomenon, the so-called constituents (Dahlberg et al., 2008). Quotations are given to illustrate the participants’ lived experiences. Where parts of a longer quotation are excluded, these are identified by a double oblique stroke (//).

The results of the analysis were processed by all authors before the final version was defined to ensure its trustworthiness. A professional English-speaking native-language editor has checked the language.

### Ethical considerations

The study was approved by the Regional Ethical Review Board in Gothenburg, Sweden (Dnr: 591–15), and conducted in accordance with the Helsinki Declaration (World Medical Association, 2013). The participants were assured of the confidentiality of their participation and that their integrity and identity would be protected. They were informed that their participation in the study was voluntary, and that they could withdraw or discontinue the participation at any point without explanation. All the participants signed a consent form. The participants received no payment to participate in the study.

## Results

The meaning structure of the phenomenon sexuality and childbearing as experienced by women living with HIV in Sweden will first be presented in its invariant part, the essence, followed by a description of four identified constituents which further describe the variances within the phenomenon.

The essence of the phenomenon is that *perceptions about HIV and its contagiousness profoundly influence sexual habits and considerations in relation to pregnancy and childbirth*. The perceptions about the potential risk of transmitting HIV, along with its severe consequences, permeate thoughts, expectations, choices, decisions and actions related to sexuality and childbearing. HIV, sexuality and childbearing are intertwined, as HIV can be transferred through sexual intercourse or childbearing, making the risk of transmission present in every act related to sexuality and childbearing. Despite the risk of sexual transmission being low, the risk of transferring the virus to a partner or child creates feelings of fear and insecurity in relation to sexual habits, pregnancy and childbirth. In addition, the perceptions of HIV infection and its contagiousness are also present among members of society and health care professionals, thereby influencing the women. The perceptions are thus formed by a combination of knowledge about HIV and interpretations by the woman herself, by those with whom she has a relationship and by those in her environment. The perceptions profoundly influence and make the woman dependent on her own knowledge, but also on her environment’s knowledge about HIV and transmission in her decision-making in relation to sexuality and childbearing. To have perceptions about being contagious, which means to see oneself as a person who can transfer HIV, places a sense of great responsibility on the woman, both juridical and morally, in relation to sexuality and childbearing.

Another aspect of sexuality and childbearing is that the perceptions of being contagious are experienced as being limiting, described through feelings of not having opportunities to choose freely how to perform sexual activities, to freely choose a partner, or not being able to choose if, when and how to become pregnant. The lived experiences of sexuality and childbearing are also shown through the relationship between perceptions about HIV and its contagiousness and knowledge about the risk of transmission of HIV. The woman is dependent on her own knowledge about the risk of sexual transmission of HIV, but also on the knowledge of others in her environment, such as a partner or health care provider. Deeper knowledge also seems to work against stigmatization and its negative consequences in relation to sexuality and childbearing. A deeper understanding and higher knowledge of HIV and risk of transmission provides a basis for making secure choices and decisions in relation to sexual habits, pregnancy and childbirth. By re-creating sexuality and childbearing in a process of learning how to cope with the virus, the perceptions of being contagious tend to have less influence on the woman’s sexuality and childbearing.

Here follows a further description of the phenomenon by its constituents: Risk of transmission imposes demands on responsibility; The contagiousness of HIV limits sexuality and childbearing; Knowledge about HIV transmission provides confident choices and decisions; and To re-create sexuality and childbearing.

### Risk of transmission imposes demands on responsibility in relation to sexuality and childbearing

One aspect of the perceptions about HIV and its contagiousness is that the risk of transmission imposes both juridical and moral responsibility in relation to sexuality and childbearing. The juridical responsibility is based on the risk of transmission and the severe consequences of an untreated HIV infection. The regulations relating to HIV are described by the women as existing for a reason but are experienced as placing the responsibility for the transmission of HIV on the person living with HIV, which seems to generate feelings of guilt and shame. The findings suggest that feeling secure and minimizing the risk of transmission can motivate women to follow regulations. Putting the baby at risk of HIV transmission, not being allowed to breastfeed due to the regulations, or being on medication while being pregnant, can create feelings of having a bad conscience among the women. It is recognized, however, that it is a logical decision to prioritize the child’s health in order to prevent transmission to the baby, even though this is sometimes challenging. As one participant described: “It was a very, very big challenge, because I had to decide. If I hadn’t got pregnant, I wouldn’t be on medication.//I started medication because I got pregnant” (IP 17).

Besides being presented as having a legal responsibility, the women experienced a sense of moral responsibility expressed as a strong desire to not transfer the virus: “When you are infected with HIV you have a responsibility not to transmit to someone else.//I feel an enormous responsibility” (IP 16).

The strong will to protect others from the transmission of HIV and that they themselves may be possible transmitters can lead to pushing people away: “I tell him … I push him, you must go.//I don’t want to kill somebody else” (IP 15).

The importance of disclosing the HIV diagnosis to a partner is therefore a way to give a partner the possibility to choose whether to be exposed to the risk of HIV transmission. This desire to disclose also means being open and honest: “I am thinking that if you want to know each other then all parts need to know, you can’t lie to each other, that’s how I am thinking” (IP 6).

Although it seems to be important to be open about the diagnosis, it also seems to be a relief to no longer have the legal duty to inform anyone about the HIV infection, illustrated through such expressions as “I feel free” (IP 3). However, not telling anyone about the diagnosis can lead to feelings of “living a secret life” (IP 4) and promotes feelings of hiding an important part of your life to the people you care about.

### The contagiousness of HIV limits sexuality and childbearing

Another aspect of the experiences about HIV and its contagiousness is that the contagiousness of HIV limits sexuality and childbearing and therefore influences sexual habits and considerations in relation to pregnancy and childbirth. The limitations are expressed through feelings of not having the possibility to choose freely in relation to sexuality and childbearing. HIV seems to have an extra dimension, its potential contagiousness, making HIV difficult to compare with other chronic diseases, as described by IP 8: “They often compare it [HIV] with diabetes.//But a patient with diabetes is not being seen as a source of infection or limits you from having sex or being close to people physically.”

HIV seems to have an impact on the women’s sexuality in negative ways. It changes a woman’s sexuality as it was known to her, and sometimes also changes her plans and considerations about motherhood:

“My sexual life is destroyed.//I won’t be able to have more children, because who is going to make me pregnant?//But to tell my partner, that I have this disease …//I think all this has limited my life mostly” (IP 3).

Expectations about people’s reactions and bad experiences of a partner leaving after learning about their partner’s positive HIV status can create a fear of being judged or rejected. This fear of being rejected, in association with the obligation to inform others about HIV, can create feelings of being limited to freely choose a sexual partner. IP 5 shares this feeling: “I don’t think that there is someone who can love me for who I am … it feels really difficult. I’m thinking I will be older and alone.”

Feelings of being unwanted and imagining that it is impossible to find a partner sometimes has the result that the woman chooses to stay in unhealthy relationships, such as abusive relationships. To disclose an HIV status to a present partner or a future partner can also bring the risk of feeling dependent, signifying the loss of power in a relationship. This feeling of being dependent is described as:

If you meet someone, and when you tell, they either run as fast as they can, or they stay. But then it feels like I’m dependent. Like they can demand things from me, like: I’m still staying with you even though you have this disease. (IP 2)

Fear of transmission makes it more difficult to relax in sexual encounters, illuminated as “you can’t relax 100%” (IP 4). HIV can be seen as a “third partner in a relationship” (IP 2), and the virus is described as something that “exists in the body”, creating feelings of being “filthy and disgusting” (IP 11). Feelings of guilt and shame seem to reduce sexual desire and there were descriptions of experiences of attempts to shut down/cut off sexuality completely.

Intimacy seems to be inhibited by condom use. It is not considered natural to use condom in a stable relationship and presents a lack of trust and also reduces sensitivity and spontaneity. This sense of inhibited intimacy is described as: “It’s not the same thing when you are using real protection.//The feeling. Everything gets a little bit more uncomfortable.//It’s a bit awkward” (IP 9). Using protection also creates insecurity about the chances of getting pregnant and is experienced as limiting for planning pregnancy.

Limitations in pregnancy for the women seem to exist because of the negative expectations of society, and even sometimes of some health care professionals, resulting in a sense that the women living with HIV have feelings of that they should not get pregnant and have children. But also, the pregnancy itself appears to be a serious reminder about HIV for some women, which seems to detract from some of the joy related to expecting a baby, and, as a result, some women choose not to get pregnant. There is also a feeling of receiving “unfair” (IP 7) treatment and discrimination, compared to HIV-negative women, when seeking help from the Swedish health care system, with such services as artificial insemination and adoption. The choices in relation to how to deliver the baby are experienced as being limited, as is the lack of possibility to breastfeed the baby:

It felt like a normal way of connection, the thing you are doing as a woman and as a mother you should really breastfeed. All people feel that. But I was not able to. And that was a bit sad. It was a loss and even vaginal birth, believe it or not, I feel like it is something I would have liked to experience. (IP 12)

### Knowledge about HIV transmission provides confident choices and decisions about sexual habits, pregnancy and childbirth

Perceptions about HIV and its contagiousness are related to an individual’s level of knowledge about HIV and risk of transmission in relation to sexuality and childbearing. A higher level of knowledge about HIV transmission provides the ability to make confident choices and decisions about sexual habits, pregnancy and childbirth. With this deeper knowledge and understanding, the woman is able to make confident and conscious decisions, and this is important in terms of feeling safe about what is and is not considered to be a risk of transmission. The level of knowledge about HIV transmission in society is considered to be low, which also affects the woman’s own feelings of confidence in the risk of transmission, expressed as:

“I can’t myself believe that the virus level is so low that the risk for transmission is so low, and when I can’t really believe in it myself …//I can’t really blame anybody else for not believing in it” (IP 14).

The general information about HIV provided to the women seems to come mainly from HIV care providers, and includes individual information about their viral load. This is described as increasing knowledge and understanding about the virus. Awareness of the effectiveness of ART and its associated lower risk of transmission is explained as having changed the whole view of HIV as a deadly disease. Not being contagious seems to bring a belief in having a future and a good life, for example: “To get this chance of seeing the doctor who says that your virus is so low so you can’t see it. That’s good. It gives me hope.//It feels safe to hear that” (IP 4).

Having a low viral load also seems to reduce the fear of transmission related to pregnancy, as expressed by IP 1: “Yeah, my child was feeling good, it was zero risk, zero percent risk for the baby. So I wasn’t actually worried.”

To hear that the viral load is suppressed and that the risk of transmission is very low, and to obtain a deeper understanding about the individual infectivity rate and risk of transmission, seems to reduce feelings of being contagious. There is a request to know more and to learn how high or low the risk of transmission is in specific sexual situations, which the health care professionals sometimes appear to have difficulties in explaining. These experiences are described by IP 14: “Yeah, he was a bit uncomfortable [the doctor] …//and he relaxes when he is permitted to talk about viral load and medication.”

Laws and regulations are sometimes questioned and experienced as old-fashioned, and requests for new scientific results are present, especially research about HIV transmission and breastfeeding: “I don’t believe you can transfer HIV through breastfeeding.//I would actually like to see a scientific study about this” (IP 11).

Information from HIV clinics and also other health care providers seems to have an impact on decision-making and brings a sense of dignity to the choices that the women make. It also affects the perceptions of HIV transmission, which places health care providers in a position of power when it comes to information about HIV, guidelines and regulations. The consequences of that dignity are described as: “It can go very wrong depending on the information you get, how you express it” (IP 12).

There are also senses or even experiences of receiving varying advice and instructions, depending on the care provider. This seems to create ambiguity and even more insecurity about the risks of transmission, for example: “Yeah, I thought it was very inconsistent and I tried to read on, tried to understand, these different guidelines for different hospitals” (IP 14).

Along with HIV clinics, the Internet is described as being an important source of information, as it offers a way to communicate with other people who are living with HIV. Also, patient organizations can be a setting for sharing and learning about other people’s experiences and knowledge. Sharing these experiences is described as being empowering; however, it also means placing yourself at risk of being exposed and having to trust other people is expressed as being difficult.

### To re-create sexuality and childbearing

By re-creating sexuality and childbearing, the perceptions of being contagious tend to have less negative influence on the woman’s sexuality and childbearing and instead strengthen her self-identity. To re-create sexuality and childbearing is about learning to cope with the virus so that sexuality and childbearing are not controlled by the perceptions of being contagious. The period of time immediately following diagnosis is described by many women as being very difficult. It seems that, as time goes by, many women are starting a process of understanding and acceptance of the diagnosis. A way to re-create sexuality and childbirth and to feel whole as a woman is to experience the joy and happiness of becoming a mother. Being a mother signifies being able to nourish and being needed, and means being part of a family. A baby can also be seen as a gift from God: “It is nothing you go and buy. It is a big gift that you are given from God” (IP 10).

Living with HIV can release feelings of alienation and a belief that one is not able to obtain what all other women have: a home, a partner, a relationship, a child, a normal life. A feeling of missing out on opportunities is common, and emotions of not being normal are described:

I was hoping I would deliver like all people … It’s important, because you have to be like other people, but this has deprived me of the chance of being like other women, so … I feel very bad, I feel very bad. (IP 18)

A supportive element that helps to re-create sexuality and childbirth can be the partner in an intimate partner relationship, and experiences of deepened relationships are expressed. A partner who shows openness and respect increases feelings of being normal, as IP 13 expressed: “I feel normal. Maybe because I have a man who understands all this and I don’t need to get reminded all the times that there is something wrong with me.”

Some women even insist that, when their partner cannot accept their diagnosis, the partner has to leave. A perceived self-confidence is expressed: “If the relationship has to work, then he has to know. He really has to know, I cannot keep him in darkness, he must know … And accept me the way I am, or leave me” (IP 17).

Having good contact with a health care provider can help the process in re-creating sexuality and childbearing. Being treated fairly and with respect are important factors in feeling normal and confirmed as a person. To be involved in treatment and care and to be able to discuss family planning or discuss contraceptives, and not to be seen only as an HIV-positive woman, compensates feelings of being vulnerable, each of which builds up the woman’s sense of self-respect.

## Discussion

The aim of this study was to describe the phenomenon sexuality and childbearing as it is experienced by women living with HIV in Sweden and it is the first Swedish phenomenological lifeworld study describing this phenomenon. The results reveal that perceptions of HIV and its contagiousness profoundly influence the sexual habits and childbearing of women living with HIV. There are different aspects of how women’s sexuality and childbearing is experienced by the perceptions about the contagiousness of HIV. The perceptions are thus formed by a combination of knowledge about HIV and interpretations by the woman herself, by those with whom she has a relationship and by those in her environment. A higher level of knowledge is important for the women to make confident choices and decisions in relation to sexual habits, pregnancy and childbirth.

Although HIV has a low risk of sexual transmission in patients treated with antiretroviral treatment (Attia, Egger, Müller, Zwahlen, & Low, 2009; Loutfy et al., 2013), the analysis illustrates that despite this, many women still experience insecurity about the risk of transferring the virus to a partner or child. After this study was conducted even more evidence about the minimal risk of sexual transmission when being well treated on ART has been published (Rodger et al., 2016). The experiences of feeling more or less contagious, the perceptions about contagiousness, create feelings of fear and insecurity about HIV transmission to a partner. This finding aligns with those of Mcgrath et al. (2014) which show that, although HIV is seen as a chronic disease, there is still stigma associated with HIV and sexual transmission. Insecurity is also experienced regarding mother-to-child transmission (MTCT), which influences women’s decisions about becoming pregnant (Craft, Delaney, Bautista, & Serovich, 2007). Our results also show that the insecurity of HIV and its contagiousness can be related to a gap in knowledge about the risk of MTCT when breastfeeding. The estimated risk of MTCT when breastfeeding is as high as between 15% to 45% when no strategies to reduce HIV transmission are taken (De Cock et al., 2000; Drake, Wagner, Richardson, John-Stewart, & Mofenson, 2014; Hira et al., 1990; Humphrey et al., 2010). More studies on breast feeding and transmission risk in women on ART are needed to close this gap of knowledge.

The first constituent in our findings reveals that an HIV diagnosis places demands on responsibility in relation to sexuality and childbearing. The strong demand for taking responsibility for the unborn baby is not only a phenomenon for HIV-positive women, but has also been found in women living with type 1 diabetes (Berg & Hotikasalo, 2000). A strong sense of responsibility has also been described in women living with chronical diseases, where pregnancy is described as a balancing act, weighing one’s own desires against the risks for both one’s own and the child’s health (Tyer‐Viola & Lopez, 2014).

The feelings of responsibility are, in our results, presented as a strong willingness to disclose the HIV diagnosis to a partner, regardless of any legal obligation. Although the legislation relating to the legal obligation to inform a partner about HIV changed in 2013 in Sweden (Albert et al., 2014; SMI, 2013), the regulations were often experienced in our findings as repressive and old-fashioned compared to those of other countries; for example, Switzerland (Hasse et al., 2010), where the law differs from Sweden in relation to the legal obligation to use a condom (Vernazza, Hirschel, Bernasconi, & Flepp, 2008). Several countries, similar to Sweden, have legal statutes relating to HIV-positive individuals about HIV non-disclosure, HIV exposure and/or HIV transmission (UNAIDS, 2012, 2013). Legal penalties for breaching these regulations contribute to the so-called criminalization of HIV and are connected with feelings of discrimination (Adam, Elliott, Corriveau, & English, 2014), and the largest study conducted in Sweden examining quality of life for HIV-positive people shows that the duty to inform affects their ability to meet a new partner (The Public Health Agency of Sweden, 2015), a finding which was also revealed in our study.

The second constituent in our results, where HIV is described as a limitation in relation to sexuality and childbearing, seems to be connected with the experiences of being stigmatized. Many studies among HIV-positive people show that stigmatization has a negative impact on quality of life (Cuca & Rose, 2016; Florom‐Smith & De Santis, 2012; Goffman, 1990; Okuno et al., 2015). Stigmatization can also increase medication adherence issues (Li, Murray, K., Suwanteerangkul, & Wiwatanadate, 2014), and can increase sexual risk behaviour among people living with HIV (Teti, Bowleg, & Lloyd, 2010; Wingood et al., 2007). In our findings, HIV is described as something that complicates, which is in line with the findings of previous research (Cuca & Rose, 2016; Grodensky et al., 2015; Ho, Goh, & L., 2017).

Stigmatization is also described as an aspect of sexuality and reproduction where the perceptions about HIV and contagiousness relate to knowledge about HIV and transmission in the people the women meet. There is a need for deeper knowledge, not only in the health care system, but also in society. Lack of knowledge generates prejudices and misconceptions about HIV and transmission. Levels of knowledge about HIV and transmission in Sweden vary (SMI, 2012), and these also seem to vary within the health care system (Hall, Plantin, & Tornberg, 2017). According to our findings, the level of knowledge about HIV is experienced as being much better at HIV clinics than for health care in general. Our findings reveal experiences of having a feeling of being questioned about the right to have a wish to become pregnant, even by health care providers, which also seems to occur in health care systems in the health care services provided in other countries (Greene et al., 2015; Kirshenbaum et al., 2004). Our findings reveal a need to provide more public information about HIV and its transmission and in that way increase the general knowledge about HIV, thereby decreasing prejudices and the stigmatization of people living with HIV. This information has to be directed to the appropriate stakeholders who hold shared interests by using the right terminologies, in accordance with UNAIDS´ Terminology Guidelines, to avoid contributing to even further stigmatization about HIV (UNAIDS, 2015) as the first media reports about HIV did in the 1980s (Reinius, 2018).

The negative effects of stigmatization in the Swedish context have to be further investigated to see whether there are differences between groups of people living with HIV in Sweden, such as gender, age or cultural background, and also how they affect sexuality and childbearing and, in the long term, health and well-being for individuals living with HIV. Furthermore, it is also important to investigate the level of self-perceived stigmatization in the health care system as a grounds for providing equal care.

The last constituent is about re-creating sexuality and childbearing, which goes in line with the view of the relation between sexuality, childbearing and psychological factors related to HIV (Bova & Durante, 2003; Siegel, Schrimshaw, & Lekas, 2006; WHO, 2013). Our study shows that many of the women experienced negative changes in their sexual lives when being diagnosed with HIV, a finding in line with another recent Swedish study examining HIV-positive people’s sexual satisfaction. (Schönnesson et al., 2018). The women in our study also experienced a change of their sexual identities in, for example, being a mother or a partner. This has also been described in other health conditions; for example, women with breast cancer who were not confident in their sexual identity felt vulnerable (Klaeson & Berterö, 2008). The authors also emphasized the benefits of an intimate relationship and the need for awareness of these issues by health care personnel (Klaeson & Berterö, 2008).

Our results show how health care providers can have an impact on feelings of acceptance, and the women in our study highlight the importance of maintaining a good relationship with a health care provider. However, the findings in our study also show a sort of ambiguity between the need and importance of providing specialized HIV care and the need to be treated in the same way as women who are not living with HIV. There is a feeling of knowing that they are dependent on the health care providers, which creates an added vulnerbility. Receiving supportive care from knowledgeable providers without feeling labelled has been shown to be crucial in promoting HIV-positive women’s adherence to treatment (Bharat & Mahendra, 2007), but it is also important to promote their sexual and reproductive health (Greene et al., 2015). The desire to talk more about sexuality and childbearing was present in our findings, a desire which has been shown in other settings as well (Finocchario-Kessler et al., 2012). Our findings illustrate that it is sometimes difficult to discuss sexuality, which seems to be a problem in general in health care services (Saunamäki & Engström, 2014). However, by discussing viral load, transmission, sexual habits, and the family planning and fertility desires of women living with HIV, care providers can influence women’s decisions to become pregnant (Jones et al., 2016; Saleem, Surkan, Kerrigan, & Kennedy, 2016). A Canadian study also revealed a connection between sexual abstinence and not having discussed viral load and HIV transmission risk with a health care provider (Kaida et al., 2015). Therefore, health care providers must integrate sexuality and childbearing as essential components in clinical assessment for women living with HIV in order to improve their health care and well-being.

It is important to pay attention to the women’s increased vulnerability due to their HIV and to empower the women so they can make safe decisions about sexuality and childbearing. This includes providing education and information about HIV and its transmission in relation to sexuality and childbearing. Moreover, because behavioural, psychosocial and health care factors can have a profound effect on pregnancy outcomes, partners should be encouraged to be actively engaged so that women’s sexual and reproductive health and rights can be strengthened (Jones, Chakhtoura, & Cook, 2013).

### Methodological considerations

In a study that performs reflective lifeworld research based on phenomenology, it is important to reflect upon the variation of experiences within the explored phenomenon. Women living with HIV in Sweden represent a very varied group of different ages and cultural backgrounds. The 18 women included in our study were strategically chosen to represent a broad variance in experiences. We included women who had not lived in Sweden for a long period of time, and, although three interviews were conducted in English, they were assessed to be rich in meanings, describing the lived experiences of the phenomenon. It is a strength of our study that we included so many women who were not born in Sweden, a strategy which therefore reflects a broad variance of the lived experiences of the explored phenomenon.

Intersubjectivity has been described by Husserl (1977) and Merleau-Ponty (2012) as a world that is individual but at the same time shared. Our results reflect this world of intersubjectivity in a very concrete way, where the women’s experienced perceptions are formed by the women’s own embodied knowledge about HIV and transmission, and which is experienced by the perceptions that exist in the relation between the woman and herself and in her relation to others.

We conducted the study by using the methodological principle of bridling to establish objectivity and validity throughout the research (Dahlberg et al., 2008). In this study, all the authors participated in discussions and reflections to reach a deeper sense of the phenomenon and of the significance of the patients’ experiences. Despite living in the era of effective antiretoviral treatment that makes the risk of sexual transmission minimal, the women were still so greatly affected by the feeling of being contagious. The use of the word “contagious” should also be avoided, due to the risk of contributing further to stigmatization about HIV and transmission (UNAIDS, 2015), but this was the word used by the participants themselves and therefore the authors chose to use it in presenting the results, reflecting the lived experiences expressed by the women themselves.

A limitation of the study is that no women were included who originated from Eastern Europe, Australia or the American continent, nor did we include any women with a history of drug abuse. However, this can be explained by the very low prevalence of patients in Sweden who originate from these parts of the world, as well as a very low prevalence of intravenous drug users in the Swedish HIV cohort.

## Conclusion

The study shows that women living with HIV are profoundly influenced by their perceptions of HIV and its contagiousness. These perceptions about contagiousness permeate thoughts, expectations, choices, decisions and actions related to sexuality and childbearing. The perceptions are also present among members of society and health care professionals, thereby influencing the women’s own perceptions. Although there is a minimal risk to transmit HIV sexually to a partner or to a baby if a person with HIV is well treated, the women are still affected by this risk, making them feeling more or less contagious. Having knowledge about the contagiousness of HIV makes it possible to estimate the risk of transmission and provides a basis for making confident and conscious choices and decisions in relation to sexual habits, pregnancy and childbirth. This is the beginning of a re-creating process about how to cope with the virus so that sexuality and childbearing are not controlled by the perceptions of being contagious. There is a need to enable and address issues connected with sexuality and childbearing to empower and encourage women living with HIV to be actively engaged in making confident choices and decisions about sexual habits, pregnancy and childbirth. By providing education and non-judgmental information about HIV and its contagiousness to women, while also disseminating knowledge about HIV and its transmission in wider society and health care, the sexual and reproductive health and rights of women living with HIV can be strengthened.
